# The Role of Micronutrients and Toxic Metals in the Management of Epidemics in Cambodia

**DOI:** 10.3390/ijerph182111446

**Published:** 2021-10-30

**Authors:** Thomas Murphy, Kongkea Phan, Kim Neil Irvine, David Lean

**Affiliations:** 1 Faculty of Science and Technology, International University, Phnom Penh 12000, Cambodia; kongkeaphan@gmail.com; 2 Faculty of Architecture & Planning, Rangsit Campus, Thammasat University, Khlong Nueng 12121, Thailand; kim.irvine@ap.tu.ac.th; 3 Lean Environmental, Apsley, ON K0L1A0, Canada; drslean@gmail.com

**Keywords:** pandemics, Cambodia, COVID-19, zinc, arsenic, mercury, micronutrient deficiency, anaemia, immunity impairment, zinc deficiency, phytic acid

## Abstract

The illegal trade of wildlife in SE Asia has been identified as the likely cause of the COVID-19 pandemic. We reviewed 198 papers on the current COVID pandemic in Cambodia, diseases such as avian influenza and Nipah virus, most likely to develop into a new pandemic in Cambodia, and common features of disease that require mitigation. Artisanal goldmining uses pure mercury in the areas where wildlife is smuggled to China. Moreover, 30–40% of Cambodians are zinc deficient. High levels of arsenic in irrigation water (>1000 µg/L) are associated with very low levels of zinc in rice (5 µg/g) and rice is the primary staple food for the region. Brown rice from nine of 15 paddy fields in the arsenic zone of Cambodia had double the new guidelines of 100 µg/kg inorganic arsenic for children’s food in the EU and USA. The combination of deficiencies of essential micronutrients like zinc and pervasive presence of arsenic and mercury has the potential to compromise the immunity of many Cambodians. Innovative solutions are suggested to improve micronutrient nutrition. Toxins that suppress the immune system must be better managed to reduce the virulence of pathogens. Cambodia was not likely the source of the COVID-19 but does have problems that could result in a new pandemic.

## 1. Introduction

**Figure 1:**
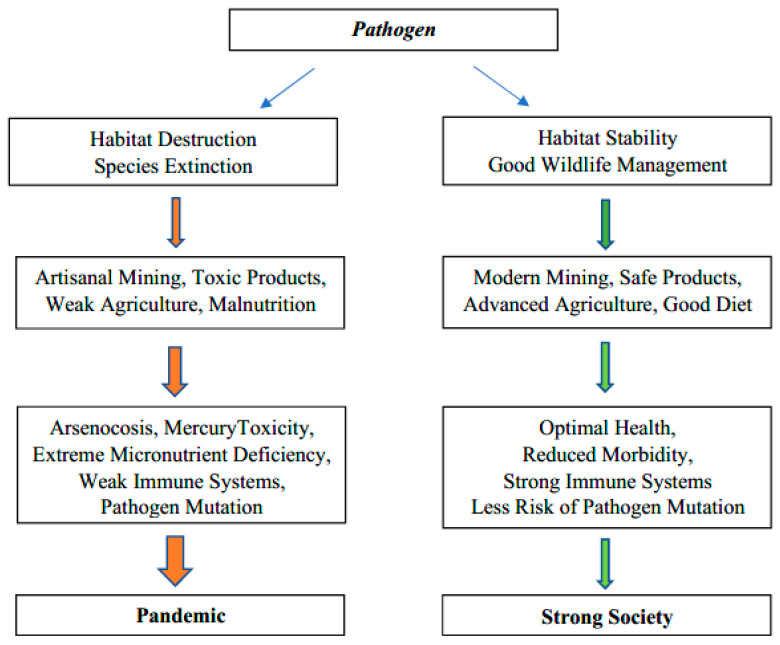
Graphical abstract: pathways to weak or strong ecosystem health.

**Figure 2:**
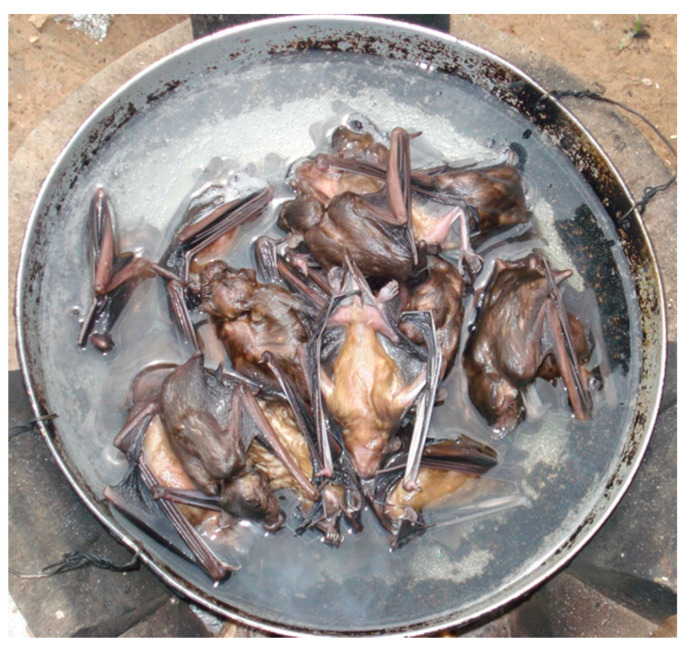
Bat lunch, Samraung Ta-ay, Kandal province, (T, Murphy).

**Figure 3:**
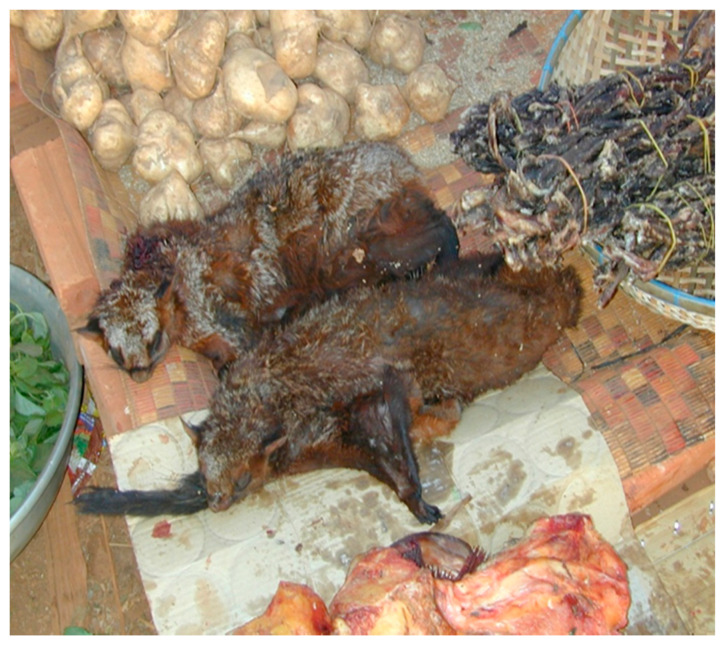
Flying fox bat (center), parrots (right) in market, Prey Meas goldmine, Ratannikiri province (T. Murphy).

**Figure 4:**
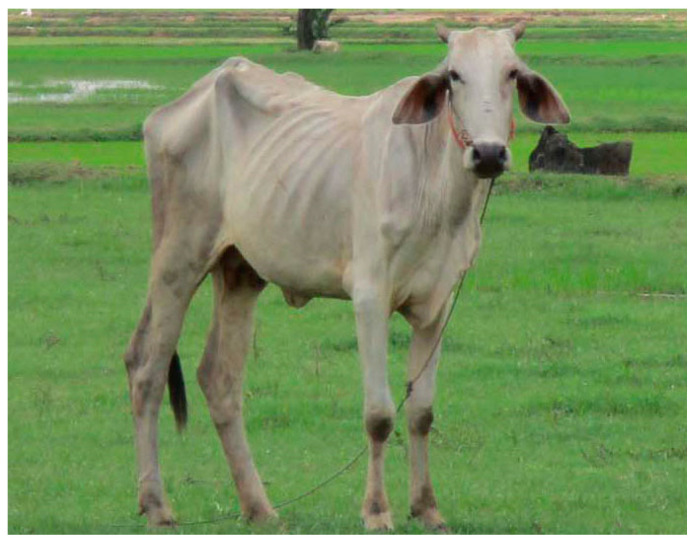
Gaunt cow, Preak Russey, Cambodia (T. Murphy).

A little less than half of Cambodia remains undeveloped, but deforestation has been rapid [1]. Habitat destruction and simplification of ecosystems enhance diseases (Figure 1), [2,3,4]. The population (16,718,965; 2020) and economy of Cambodia (GDP per capita $1215; 2020) are growing rapidly. Urbanization is proceeding quickly and partly reflects the rapid development of garment factories [5,6]. There are significant differences in the nutrition of urban and rural Cambodians but recent migration probably reduces the extent of regional differences [7]. The greater presence of stunting of children in rural areas seems to reflect a greater deficiency of zinc [8,9]. Over a million young Cambodians, many from rural areas, have left to work overseas, mostly in Thailand [6]. Moreover, several hundred thousand young Cambodians have migrated to Khmer cities, particularly Phnom Penh, in the search for work [6,10]. These migrations result in the farms having too little labour for traditional farming methods. They also result in many young adults from rural areas living in crowded areas near factories which is where the hot spots of the COVID-19 developed. Many farms are still small and simple. Many Cambodians want their own land and those working overseas in Malaysia and Thailand send an average of $1800 US a year home, often to maintain small farms [6]. In parallel, plantations and other forms of agribusiness are changing social structure and farming practices significantly [11]. A common unifying theme of all Cambodians is that rice is a dietary staple, but one which in some locations is deficient in zinc, one of the most important micronutrients for good health and effective immune systems [12].

Although Cambodia is relatively close to the source of the COVID-19 pandemic, for the first year of the pandemic, it was able to escape widespread transmission with a total of only 366 cases by 31 December 2020 [13]. Quarantines, monitoring, social distancing, use of masks, and community tracing were effective until four foreign nationals escaped quarantine and subsequently became superspreaders [14]. During the pandemic, the arrival of international tourists fell by nearly 80% with the worst months having declines of 99% [15,16]. At the time of the major COVID outbreak in Cambodia (20 February 2021), fewer than 1% of people had two vaccinations [17]. Vaccines purchased from or donated by China, and 10% donated by the World Health Organization COVID-19 Vaccines Global Access, (WHO COVAX) [18] enabled Cambodia to attain the second highest percentage of population vaccinated in Association of Southeast Asian Nations (ASEAN) by May 2021. But the outbreak continued vigorously [19]. Analysis using molecular clock phytogenetic analyses indicated that the COVID-19 pandemic developed in China within weeks before it was detected [20]. The development of vaccines happened with amazing speed but not quickly enough to prevent massive suffering. By October 2021, the COVID outbreak in Cambodia was mostly contained; vaccinations greatly enhanced the ongoing government management [19]. In the future, every possible action to minimize the risk of pandemics must be at least considered even if they can only alleviate some morbidity. 

Comorbidities have been implicated in many COVID deaths [21]. Upon hospital admission, 50% of patients have been reported with at least one comorbidity with COVID-19 [22]. Hypertension, diabetes, chronic obstructive pulmonary disease, obesity, and cardiovascular diseases are the most commonly reported comorbidities [22]. This wide variety of comorbidities with COVID-19 and observations that environmental factors influence COVID-19 [23,24] suggests there will be other comorbidities of importance in COVID-19 development. Toxic metals have been linked to induction of the COVID but not yet clearly demonstrated by clinical evaluations [25]. In the countries with the worst metal contamination, COVID greatly suppressed the medical system. Alternative strategies are needed, beyond clinical trials to resolve comorbidities. In North Carolina University, researchers are using bioassays with genomes bioengineered to contain human genes to evaluate the basics of arsenic mediation of COVID-19 comorbidity [26]. Such tools might also provide clarity for therapeutics. Of course, we acknowledge the importance of vaccinations and therapeutics, but avoidance of pandemics requires more innovative, strategic evaluations, planning, and management. We will review examples, mostly in Cambodia where toxic materials and nutrient deficiency weaken the immune systems. Management strategies for pandemics should focus on the major problems like arsenic and mercury toxicity, and zinc deficiency in food. 

The worst cases of metal toxicity suppressing community health often are found in developing countries with limited resources for clinical studies. Before the COVID-19, in Bangladesh 20% of deaths were related to arsenic from tube wells [27]. While we recognize that elevated levels of metals such as manganese (e.g., in drinking water), cadmium (e.g., in some poorly produced agricultural fertilizers), and lead (e.g., in household paints) have the potential to impact community health in Cambodia, arsenic is by far the most important toxic metal in food and water in this country [28,29,30,31] and globally [32,33,34,35]. Arsenic impedes the immune system [36] but a linkage of arsenic toxicity with COVID-19 has not been resolved. We will discuss methods to improve arsenic management and in turn strengthen immune systems thereby potentially increasing resilience to pandemics. 

Mercury is a very common toxic metal throughout the world [37,38,39]. In Cambodia, mercury is very commonly found in skin whitening creams, at levels of up to 3.5%, and in traditional Chinese medicines at levels of up to 82% [39]. Mercury complexes and inactivates selenium, an essential micronutrient in immune systems [40,41,42]. This concept of a toxic metal inactivating an essential micronutrient is similar but better understood than the apparent ability of arsenic to impede assimilation of zinc in rice [28]. Most people do not develop severe cases of the COVID and the typical immune response is sufficient to overcome the virus [43]. Furthermore, many of the factors that result in COVID-19 becoming more deadly are also commonly associated with mercury toxicity; diabetes, age, gender and obesity [43]. 

With respect to the COVID-19, Sethuram et al. [44] stated that “In the general population, zinc supplementation could be beneficial in both enhancing immunity and in fighting against the viral disease process.” Others have made similar suggestions [45,46,47,48] but this potential utility cannot be overstated as treatment especially in the later stages of the COVID disease. Zinc was evaluated in four treatment programs and shown not to be effective once the disease has progressed to the cytokine storm [49]. Zinc supplementation “might be a useful tool in preventing progression of the disease” [49] and should be evaluated for its potential to reduce initial infection. As with metal toxicity, the worst cases of micronutrient limitation occur in developing countries and studies on zinc augmentation of the diet in the United States, where there typically is not a zinc deficiency problem, are less relevant for a country like Cambodia where zinc limitation is associated with stunting of 40% of children [50].

This paper focuses on how agricultural methods in Cambodia could be improved to reduce zinc deficiency and in turn enhance the immune systems in Cambodians and their livestock [51,52,53]. We also review diseases in Cambodia with the potential to cause another pandemic. Better agricultural methods should improve the health of most Cambodians whereas alternative strategies such as post-harvest supplementation of food is more relevant to urban populations. Post-harvest augmentation of human diets is best provided to children in schools in that multiple nutritional issues can be targeted [54,55,56,57] and it has the additional benefit of encouraging children to stay in school. Cambodia needs to improve its management of arsenic, mercury and zinc primarily for a strong healthy population but in doing this, the chances of pandemics developing should also be significantly reduced. These strategies are relevant to many similar countries.

## 2. Methods

A literature search found >28,500 publications of relevance to our interests that were identified through ResearchGate, Hinari, Agora, Oare, Google, ScienceDirect, PubMed, and Scopus using the key words pandemics, Cambodia, immune system, COVID-19, zinc, arsenic, malnutrition, micronutrient deficiency, anaemia, stunting, mercury, immunity impairment, zinc deficiency, phytic acid, phytase, livestock micronutrient deficiency, and fish micronutrient deficiency. These publications were grouped according to the major themes of our review (e.g., zinc deficiency in humans; arsenic concentrations in rice). Subsequently, we conducted a detailed assessment of 198 papers, based on exceedances of international guidelines and documented history of impairing the immune system. Arsenic and mercury were the most common toxins inhibiting immune systems and zinc was the common micronutrient deficiency inhibiting immune systems. 

## 3. Discussion

### 3.1. Factors Regulating Pandemics and Their Potential Management

#### 3.1.1. Human Exposure to Wildlife and Livestock Diseases

One of the theories for the initiation of the COVID-19 pandemic is the illicit trade in animals captured in the forests in SE Asia with export via Vietnam and their rearing in cages in close contact with humans in China [58]. A coronavirus isolated from a bat in Cambodia contained 92.6% of the same nucleotides as COVID-19 [59]. Almost every gardening center in Cambodia sells bat guano. Often bat guano contains histoplasmosis, a serious and difficult to treat fungal disease in humans [60]; the bat guano also contains other pathogens [61]. Specifically, bats are known to transmit many viruses, some of which are extremely pathogenic in humans: henipaviruses (Nipah and Hendra), filoviruses (Ebola and Marburg), and coronaviruses (SARS-CoV and MERS-CoV) [62]. Flying fox bats (*Pteropus vampyrus*) are the primary reservoir of the Nipah virus in Cambodia; an outbreak of this virus in Malaysia resulted in 105 deaths in 265 infections (40% fatality), [63]. Flying fox bats are used as bushmeat, food neophilia and in traditional medicine in Cambodia [64] and China [65]. The Nipah virus (NiV) was found in a total of 20/3157 (0.63%) and 8/773 (1.03%) flying fox bat urine samples in Kandal and in Battambang, Cambodia, respectively [66]. The contamination of palm juice by saliva or urine from flying fox bat has been blamed for human deaths from the Nipah virus in Bangladesh [67].

Figure 2 illustrates bats being boiled for lunch in a farm near Samraung Ta-ay, 40 km S.E. of Phnom Penh on Highway 1, in an arsenic contaminated zone of Kandal province. Figure 3 shows flying fox bats for sale in a small market in Prey Meas artisanal goldmine, in Ratanakirri Province [68]. The parrots to the right of the bats were defeathered, dried and wrapped for transport (Figure 3). Parrots are expensive and gold miners could not afford to buy these birds; they were likely prepared for export. Prey Meas mine (13°31′22.9′′ N, 107°22′46.6′′ E) is only 40 km from the Vietnam border. At the time of this picture (2006, Figure 3), there were large ongoing shipments of lumber to Vietnam, apparently illegal. In 2013, a major Cambodian newspaper reported that the local use of flying fox bats in traditional medicine included drinking the bat blood [69]. Riccucci [65] reported that the drinking of bat blood was common in traditional medicines in many cultures. Another common pathway of virus transmission is via ectoparasites on bats [62].

The sales of such traditional medicines from wildlife in Cambodia are usually made in a clandestine fashion [64]. Riccucci [65] reported that 40% of the population in China used traditional medicines. Riccucci [65] also stated that a Vietnamese pharmacy imported the faeces of *Rhinolophus* bats, in batches of 40 tonnes. The common exposure to bats in SE Asia, including in household plant fertilizers and traditional medicines, could have produced some immunity and resulted in the year-long delay in the initiation of the COVID-19 outbreak in SE Asia. Traditional medicines may at times produce a good effect but they also can be dangerous both for what they are and how they are prepared [70,71]. The people who smuggle animals for traditional medicines can also be dangerous especially to those trying to suppress illegal trade. In these areas, an off-duty policeman is recommended as a driver. Visits must be announced in advance and borders areas should be avoided. 

Another potential route of virus diseases is the eating of rats collected from rice fields in Cambodia. The rats are usually kept alive in cages until prepared for cooking. Several diseases are spread by rats in SE Asia: haemorrhagic fever with renal syndrome (HFRS), leptospirosis, bartonellosis, trypanosomosis, and babesiosis [3]. In Vietnam, Huong et al. [72] used polymerase chain reaction (PCR) to detect coronavirus sequences in field rats (34.0%, 239/702) destined for human consumption and insectivorous bats in guano farms (74.8%, 234/313) adjacent to human dwellings. Avian influenza commonly kills poultry, and humans in close contact with animals can catch this disease. It is rarely transferred from human to human but it is deadly when it does infect people. With clade H5N1, 455 of 862 (53%) patients died and with clade H7N9, 616 of 1568 (39%) patients died [73]. Avian influenza has been evolving to form several variants or clades and with the right mutation, it could become more transmissive in humans potentially resulting in a very deadly pandemic [73,74]. In simple farms, chickens and pigs often roam freely, a practice that should be discouraged by through educational outreach or replacement with more professional farming. Swine flu is yet another example of an animal disease, still a concern in Cambodian agriculture and that did become a human pandemic in 2009 [75]. Now it is mostly contained but is still a risk to people with weak immune systems. Furthermore, Cambodian cattle sometimes succumb to foot-and-mouth viral diarrhea virus (BVDV). Figure 4 shows a gaunt cow in Preak Russey, Cambodia an area with high arsenic and low zinc, both impairing the immune system [12].

#### 3.1.2. Factors Enhancing the Transmission of Diseases in SE Asia

##### Habitat Degradation

Disease can be enhanced by ecosystem instabilities. In the Great Lakes of North America, outbreaks of avian botulism used to happen once in 10 years. But with instabilities introduced by invading species like zebra mussels and toxic algae, avian botulism reoccurred yearly for several years [2]. Climate change is imposing serious problems with weather instability in SE Asia that has resulted in more intense rainfall and intense droughts [76]. The large changes in agriculture in the Green Revolution also imposed widespread “*epidemics of micronutrient deficiencies*” [77]. These micronutrient deficiencies would have enhanced disease through suppression of the immune system. As habitat is simplified by human development the diversity of pathogens on rats decreases but the risks of disease transmission increases [3]. Although domestic poultry have been the largest source of avian influenza, the release of wild birds in religious acts or by transmission from wild birds in ‘wet markets’ were important initiators of outbreaks of avian influenza [4]. Two of the most extreme examples of habitat destruction in Cambodia directly compromise immune systems are artisanal gold mining using mercury and irrigation of rice with groundwater rich in arsenic.

### 3.2. Toxins in the Environment

#### 3.2.1. Mercury

**Figure 5:**
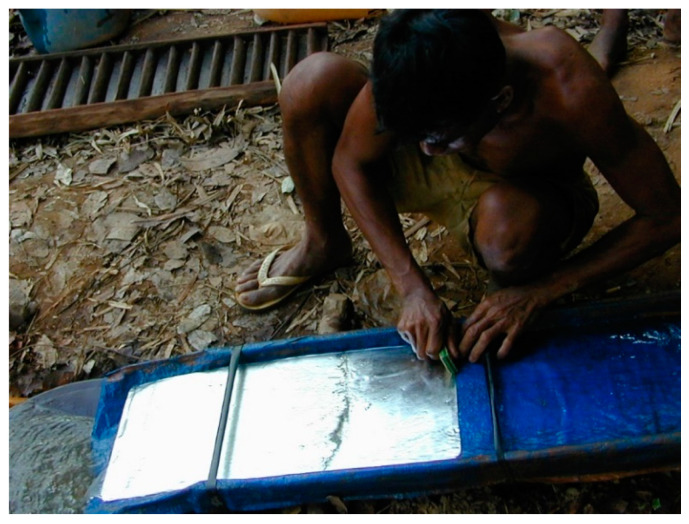
Scraping mercury with gold from flume. (T. Murphy).

**Figure 6:**
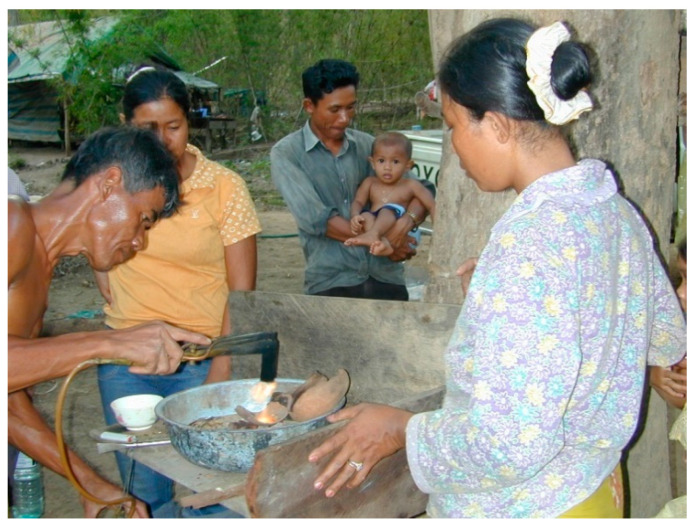
Miner separating mercury from gold (T. Murphy).

The WHO [78] advised against drinking alcohol during the COVID-19 pandemic in part because it weakens the immune system. During the worst of the COVID-19 outbreak in Cambodia, sales of alcohol were officially prohibited. Blocking other toxins that suppress the immune system is not so simple. Various forms of mercury, including inorganic mercury have been known for three decades to impair the immune system [79,80,81]. The artisanal goldminers of Cambodia are poor with inadequate diets, no medical services and frequently use pure mercury to extract gold with no safety equipment (Figure 5 and Figure 6) [39,68]. The goldminers we interviewed all said their mercury came from Vietnam [68]. Cambodia should cooperate with experienced NGOs in training artisanal miners to extract gold without mercury [82]. As noted above, artisanal goldminers work with mercury in areas where wildlife is illegally collected for shipment to China (Figure 5); this double exposure to mercury and wildlife potentially with viruses is dangerous. In the Amazon, mercury use by artisanal gold miners has been shown to increase malaria fourfold [83,84]. The use of toxic metals, such as mercury is also common in Chinese traditional medicines. As with the wildlife trade near the goldmine, the sales of traditional medicines from China containing cinnabar (mercury sulphide mineral) in Phnom Penh are often hidden but even large samples of pure cinnabar ore (77–82% mercury) are available to those that ask appropriately [39].

Agusa et al. [85] reported the enhancement of female hormones estrone and estradiol in men and women in Phnom Penh by an unknown source of mercury. This endocrine disruption by mercury most likely represented use of skin whitening creams or Chinese traditional medicines. Skin whitening creams are commonly used in Cambodia and can contain up to 3.5% mercury [86]. Skin creams contain several other toxic ingredients [86] and there are general concerns associated with the use of sun block creams. Sun blocks usually have less mercury, but the use of sun block creams prevents the body from producing vitamin D. Vitamin D deficiency also weakens the immune system. In Thailand, avoidance of the sun and use of skin creams resulted in 95% of nurses being deficient in vitamin D [87]. Vitamin D deficiency is common in Cambodian women, even in rural areas [88]. If people cannot expose their skin to the sun 20 min a day and accept a deeper skin tone, vitamin D supplementation is required. 

Fish are another potential source of mercury. Studies in Hong Kong have observed a reduction of fertility in men who ate large predatory fish rich in mercury [89]. The mean levels of mercury in human hair in a fishing community in Northern Cambodia (4540 ± 810 µg/kg, *n* = 25), were virtually the same as the level of mercury in hair associated with impaired male fertility in Hong Kong (4233 µg/kg, *n* = 117) [68,89]. Mercury is commonly found in high concentrations in predator fish such as barracuda and shark but there are little such data in Cambodia [39,88]. In much of the developed world, mercury in fish is managed with fish consumption guidelines but these have not been developed in SE Asia [90]. 

Mercury in the worst of the skin creams and traditional medicines will inactivate selenium in the human body and result in severe deficiencies of this essential micronutrient [41,42,43]. Selenium is essential in several aspects of the human immune system [91]. Selenium has been used to inactivate mercury but the change from an essential micronutrient to a toxic metal happens quickly with selenium. Many agencies avoid this remediation method because it is potentially dangerous [92]. Fortunately, unlike selenium, zinc is only toxic at very high concentrations [93]. Interestingly selenium has been used experimentally to reduce the toxicity of arsenic [94] and cadmium [92]. It is better to avoid arsenic and mercury toxicity than to treat individuals, post-exposure. Especially relative to arsenic in drinking water, cadmium appears to be a minor problem at least in Cambodia, albeit there is not much data on cadmium [95,96]. There are fears that the concentration of cadmium in phosphate fertilizers will increase as the best sources of phosphate rock are depleted [95]. Furthermore, if agricultural practices in rice cultivation were modified to produce an oxic environment in soils, cadmium would be more bioavailable, albeit today most rice is grown in reducing environments [95].

#### 3.2.2. Arsenic

Mercury and arsenic have been associated with an increased risk of respiratory dysfunction and disease, and may also play a role in COVID-19 infections [25]. There are over 2.4 million people living in the arsenic contaminated zone of Cambodia [89]. Arsenic impairs the immune system; thus, the potential for disease to spread is likely enhanced in Cambodia [97]. The introduction of tube wells in much of Asia exposed millions of people to arsenic toxicity. Graziano [27] concluded that 20% of deaths in Bangladesh were related to arsenic from tube wells.

Eliyan et al. [98] found that 6.3% of farmers in Kandal, Prey Veng and Kampong Cham Provinces, in Cambodia drank water with more than 50 µg/L or arsenic which is the Cambodian guideline for drinking water. The WHO guideline for arsenic in drinking water is 10 µg/L [99]. Dr. Mickey Sampson, a pioneer in modern Cambodian environmental science and his NGO, Resource Development International (RDI) found areas along the Mekong River with groundwater containing more than 3000 µg/L of arsenic [100]. These findings were validated by international efforts that included Columbia University [101]; EAWAG, Switzerland [102]; Environment Canada [103]; Gwangju Institute of Science and Technology, S. Korea [104]; Manchester University, UK [105]; and Stanford University, USA [106].

In Cambodia, arsenic toxicity can be as subtle as impairment of intellectual development, but may also include congenital birth defects in children [107]. In adults, arsenic toxicity may lead to amputation of cancerous limbs and mortality [108]. The inorganic arsenic content in drinking water from Preak Russey, Cambodia (23 wells 883 ± 332 µg/L) was the most important source of arsenic in the human diet [109]. The provision of arsenic-free drinking water in 2015 to Preak Russey removed about 95% of the dietary arsenic exposure [109]. Prior to 2015, drinking water was a 23-times greater source of inorganic arsenic than rice. This delivery of arsenic-free water was essential but there are still concerns with arsenic. For example, brown rice from nine of 15 paddy fields sampled in Preak Russey had double the new guidelines of 100 µg/kg inorganic arsenic for children’s food in the EU and USA [110,111,112]. 

#### 3.2.3. Management and Remediation of Arsenic in Agriculture

##### Irrigation Water

Not only are there problems with arsenic in groundwater, there are concerns that within 15 years there will not be enough water in the Mekong River system to sustain current levels of irrigation in the dry season [113]. Storage of water in wetlands near rice fields is sometimes physically possible but this may raise concerns with respect to fisheries spawning and would require further assessment. Innovative and sustainable water management strategies are needed to increase the agricultural community resilience and improve food security. A large irrigation project (budget $4,898,775 US) is scheduled to be undertaken along the Mekong River in Cambodia, Vietnam, Thailand and Laos using groundwater [114]. This must be managed very carefully or similar problems will develop as happened in Bangladesh. Forty to 50 years ago, tube wells were drilled in Bangladesh by United Nations International Children’s Emergency Fund (UNICEF), the World Bank, and the United Nations Development Programme (UNDP). Initially no arsenic analyses were undertaken. In 2010, one in five deaths in Bangladesh was related to arsenic from these tube wells [27]. Cambodia has a shorter, less intense history of arsenic contamination than Bangladesh. We have seen farmers in Bangladesh and Cambodia using arsenic rich water for irrigation who knew the risks of arsenic but felt it was necessary to make a living. Management should occur before new wells are constructed. It is difficult to monitor and regulate each well once it is dug.

The guidelines for arsenic in irrigation in Australia [115] Canada [116] and EU [117] are the same at 100 µg/L (Table 1). Countries that grow more rice have lower guidelines for arsenic in irrigation water; Japan, 10 µg/L [118]; Italy, 20 µg/L [119]; S. Korea, 50 µg/L [120]; and Taiwan, 50 µg/L [121]. A lower standard for arsenic is appropriate for irrigation of rice in that rice is more sensitive to arsenic bioaccumulation [85]. In Preak Russey, the mean concentration of total arsenic in 21 field wells was 959 ± 351 µg/L or 10 times the EU and 100 times the Japanese guidelines for irrigation water. In interviews at 17 farms in Preak Russey that included 86 participants and a nearby control site in Kandal province at 11 farms and 74 participants, 41% and 42%, respectively of farmers in both areas knew nothing about arsenic [89]. This statistic and divergence of knowledge of arsenic was especially surprising in Preak Russey where because of extreme arsenic levels and obvious arsenic mediated disease, the villagers had been supplied with piped water, with 4 µg/L of total arsenic. The farmers need help to manage their arsenic exposure.

Nor is it obvious that enough effort has been expended to develop alternative crops for the dry season in Cambodia, which utilize less water and bioaccumulate much less arsenic. Rice has one of the highest demands for water and because of its long flooding, it is the worst for arsenic bioaccumulation [110]. In Pakistan, wheat alternating with rice was considered highly successful in reducing arsenic bioaccumulation while still sustaining food production [122]. To introduce something as innovative as growing wheat in the dry season of Cambodia would require a field station and financial support to demonstrate the productivity and crop quality. The lack of a buyer would be an initial barrier for wheat to be a successful crop in Cambodia; farmers often plant what their local buyers request. Beans and corn can also be grown in the dry season in Cambodia, use less water and would increase the agricultural community resiliency to climate change.

#### 3.2.4. Arsenic Treatment

In Preak Russey, Murphy et al. [96] observed removal of more than 90% of the arsenic from groundwater that was pumped into two reservoir ditches with residence times of about 1.5 to 3 days before being applied to the rice fields. The concentrations of arsenic in the groundwater in two wells had been 633 µg/L and 1044 µg/L As. In both ditches the removal of arsenic was associated with intense floating microbial mats that became coloured red from iron. Relative to the arsenic content in the groundwater the iron content of these floating mats was 14 times enriched which is inconsistent with the simple idea of iron arsenic precipitation. A likely mechanism of arsenic removal is the microbial production of trimethyl arsine gas, (TMA) [123]. These ditches were built initially to enhance drainage at the end of the rainy season but they are also used for water storage and occasionally for irrigation. This arsenic removal process is attractive in that it did not require any added chemicals and did not produce a toxic waste that required storage. The farmers used nets to keep fish out of the water pumps and the nets likely were important to increase surface area for microbial growth. The nets also retarded flushing of a surface floating microbial mat. If the pathway of arsenic removal (TMA) was validated, the process could be optimized to take less space. The variables to be optimized could include; surface area (using straw, tree branches, netting), residence time, and potentially phosphate addition; phosphorus would be retained in the irrigation water to fertilize the field at no additional cost to rice production. We will later review other agricultural methods that alleviate arsenic toxicity while also enhancing the zinc availability of soil. First, we will review the dietary requirement for zinc and the relevant biogeochemistry of zinc in soils.

### 3.3. Effect of Micronutrient Deficiency on Human Disease 

Many recent papers review the necessity for micronutrients to combat disease, especially COVID-19, with some variation in the priorities but usually accenting the need for zinc [124,125,126]. Chen et al. [127] estimated that 50% of the population in Asia is at risk of Zn deficiency. In developing countries, where there are adequate data, 50% of children are zinc-deficient [128]. Zinc plays several roles in a healthy immune system such as regulating neutrophil extracellular traps which can capture pathogens, regulating inflammation, and controlling oxidative stress [129]. Hundreds of treatments with zinc have been studied and reported. The three largest reviews of augmentation trials have shown significant reduction of disease by zinc supplementation but there have been some deviations [130,131,132]. Mayo-Wilson et al. [131]) summarized 80 randomized controlled trials with 205,401 participants. Both Brown et al. [130] and Mayo-Wilson et al. [131] found significant reductions in diarrhoea-, pneumonia-, and malaria-related morbidity related to zinc augmentation. 

Recent zinc augmentation trials with COVID-19 patients were more modest in scale but still notable. In 249 COVID-19 patients in Spain, serum zinc levels lower than 50 µg/dl at admission correlated with worse clinical presentation, longer time to reach stability and higher mortality [133]. Similarly, in India, Jothimani et al. [134] found that in 27 COVID-19 patients, 57.4% were zinc deficient, had higher rates of complications (*p* = 0.009), acute respiratory distress syndrome (18.5% vs. 0%, *p* = 0.06), corticosteroid therapy (*p* = 0.02), prolonged hospital stays (*p* = 0.05), and increased mortality (18.5% vs. 0%, *p* = 0.06). These two COVID-19 trials were small but consistent with expectations on the importance of zinc in severe cases of COVID-19 [22].

Graham et al. [77] reviewed examples, especially with zinc, iron and Vitamin A, where treating one limiting micronutrient when another was also limiting could be ineffective. The reported variability of results from zinc augmentation needs resolution but Mayo-Wilson et al. [131] felt that the benefits of preventive zinc supplementation outweigh the potential harms in areas where the risk of zinc deficiency is high. The variability in responses often reflects the co-occurrence of more than one nutrient limitation. A full review is not warranted but a few examples are relevant. 

Yakoob et al. [135] determined zinc supplementation in children in developing countries was associated with a reduction in diarrhea mortality of 13% and pneumonia mortality of 15% but had no effect on malaria mortality. Conversely, Augustin et al. [136] found that zinc and vitamin A supplementation had a major reduction of malaria morbidity in Burkina Faso. In Peru, Penny [137] estimated that zinc supplementation reduced diarrheal deaths by 13% and pneumonia deaths by 20%. Rerksuppaphol and Rerksuppaphol [138] found that zinc supplementation of 64 Thai children with acute lower respiratory tract infections resulted in shorter hospital stays (*p* = 0.008). It is possible that for Cambodia and Thailand the most important deviation from expected results for zinc augmentation was from neighboring Laos. A trial was done with 3407 Laotian children where the prevalence of stunting and zinc deficiency was 37% and 76.5%, respectively [139]. Despite improving zinc status, preventive zinc and micronutrient powder had no impact on growth of Laotian children. An additional nutrient limitation might have been missing.

### 3.4. Micronutrient Deficiency in Cambodia

The following micronutrient deficiencies in the human diet in Cambodia are common; vitamin A, iron, zinc, iodine, vitamin B1, vitamin B9 (folic acid), vitamin B12, and vitamin D [140,141]. A review of micronutrients indicated that zinc, vitamin A and vitamin D are the most important micronutrients for a healthy immune system [142]. They are all important but options for improving their management via lifestyles in Cambodia are likely best with zinc. 

#### Zinc Deficiency in Cambodia

It has been estimated that >40% of Cambodian children are at risk of zinc deficiency [50]. Wieringa et al. [9] proposed that zinc deficiency in Cambodia is partially responsible for stunting in children. Greffeuille et al. [8] stated that about 32% of Cambodian children were stunted and proposed that zinc deficiency was a major cause. The deficiency in zinc is driven mainly by two things. (1) Some Cambodians cannot afford to buy meat or fish which are rich in zinc [143]. (2) In Asia, rice is the main energy component of food, and also the principal nutritional source of dietary zinc, but rice has a low concentration of zinc [144]. The mean content of zinc in 24 samples of brown rice in Preak Russey, Cambodia was 19.4 µg/g; whereas the World Food Programme proposed that fortified rice should contain more than 50 µg/g for people eating >300 g/d of rice [145]. Post-harvest rice fortification would have some advantages such as the opportunity to better manage other deficiencies like vitamin B1 [140], vitamin A or iodine [146]. 

In extremes of extensive water stagnation or high arsenic concentrations, zinc deficiency in growing rice results in straighthead disease and is obvious by the yellowing of the rice. Zinc deficiency in agriculture, especially as related to farm productivity, plant disease, and nutrition/health/disease of livestock, should be remediated to improve the zinc content of human food. Joy et al. [147] concluded that zinc-enriched fertilizers were a cost-effective potential health intervention in Africa if human disability was considered. The concept is appropriate but the farmers must still be economically compensated if there is no benefit accrued from higher crop yield.

### 3.5. Inferior Fertilizers and Misinformation

A recent survey of more than 40 rice farmers in Preak Russey and Kandal province of Cambodia showed that fertilizers being applied to their fields did not contain significant levels of zinc [109]. Our experience with several hundred farmers in Battambang, Pursat and Kampong Cham provinces indicates that zinc fertilizers are not used in Cambodia; but they should be. With experience and appropriate monitoring, fertilizer application could be designed to minimize zinc precipitation and inactivation in fields. Excessive levels of phosphorus can interfere with zinc assimilation, yet both are essential. Fertilization must be undertaken with skill, but in Preak Russey and Kandal province of Cambodia, the phosphorus content of the Vietnamese fertilizers was often half of that shown on the package labelling [109], which presents a challenge to effective fertilizer application.

There are several conflicting reports suggesting that zinc is not deficient in agriculture in areas that medical reports indicate people are zinc deficient. This conclusion could at times be correct. As with two or three co-occurring micronutrient deficiencies in people, soils deficient in zinc can be classified into either acute deficient or latent deficient [77]. A latent deficiency is one masked by a more severe deficiency of another nutrient, often nitrogen or phosphorus, such that the latent deficiency becomes limiting after the other, more acute deficiency is corrected. Results could also be misconstrued if zinc precipitated in a long period of stagnated, standing water in the rice field [148]. Similarly, small studies within heterogenous communities having divergent lifestyles could produce human health data inconsistent with agricultural data. 

#### Alternative Fertilization

Foliar application of zinc should be effective, at least in the dry season [147]. There are uncertainties on whether foliar application will increase rice productivity but it usually increases the zinc content of rice. Without compensation for enriching their rice with zinc some farmers would not be satisfied without an increase in rice productivity.

Small farms that have cattle, use the manure on their rice fields and in Preak Russey/Kandal province. These farms with cattle had significantly more zinc in the rice [12]. However, too much manure will enhance anoxia in the rice paddies and precipitate zinc. All of the small farmers that we worked with grew their own feed and the threat of excessive manure application to such small farms is small. Moreover, use of the appropriate amount of organic matter enhances the availability of zinc and maintaining the organic content of the soils is a wise management strategy [149].

**Figure 7:**
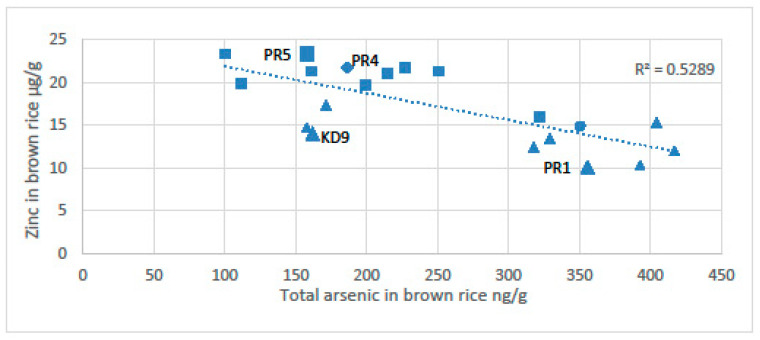
Total arsenic vs. total zinc in brown rice composite samples. [12]. Squares represent farms with cows and triangles represent farms without cows. Samples represented by the larger-sized symbols (Preak Russey-1, KD-9 and Preak Russey-5) were collected with a fixed grid of nine subsamples. The diamond represents Preak Russey-4, which had a treatment ditch.Abbreviations: Kd9, Kandal-9 farm; PR5, Preak Russey-5 farm; PR4, Preak Russey-4 farm; PR1, Preak Russey-1 farm.

The potential for chronic arsenic toxicity via rice is associated with induced micronutrient deficiency. Murphy et al. [12] found an inverse relationship between the concentration of arsenic in rice and the content of zinc (Figure 7). The anoxia associated with sustained flooding of soils precipitates zinc with sulphides and dissolves arsenic. The arsenic content of rice was highest near the wells and the zinc content of rice was highest far from the wells [12]. Near the wells the total arsenic content of the soils can be as high as 95 µg/g, which is about twice the arsenic level of the Dutch remediation guideline requiring consideration of intervention or remediation (55 mg/kg), [150]. The arsenic in the soils clearly reflects the high input of arsenic in the irrigation water (>1000 µg/L); the farmers spend time every day near the pumps maintaining them. This routine gives the farmers frequent exposure to very contaminated soils. Near the well with the most arsenic in the Preak Russey study area, the rice had only 5.7 μg/g of zinc or 10% of that recommended by the United Nations World Food Programme [6,12,145]. Similarly, Williams et al. [151] also observed high concentrations of arsenic associated with low levels of zinc and selenium in rice grain in Bangladesh. 

### 3.6. Improved Irrigation

Mid-season drainage of the rice fields is able to reduce bioaccumulation of arsenic, enhance rice productivity, increase the zinc content of rice but implementation of such drainage requires coordinated irrigation water management [96]. Any form of mid-season drainage of rice paddies could only be implemented if the farmers had a reliable source of water to reflood their fields. Another irrigation technique that should be considered is alternative wet dry (AWD) whereby the fields are usually drained twice and reflooded. Two forms of AWD decreased the arsenic content 41–68%, although there can be a modest decrease in rice yield [152].

AWD irrigation increases the zinc content while it decreases the phytic acid content of rice [153]. This change in the ratio of phytic acid to zinc significantly increases the bioavailability of zinc [154]. Furthermore, zinc fertilization of rice paddies decreased the phytic acid content of rice [153]. Phytic acid is one of the most important antimetabolites found in food. One of its metabolic functions in plants is regulating the bioavailability of zinc which is likely one of the mechanisms that mediates its ability to regulate which microbes can grow. This theme is discussed more in a section on aquaculture later. Phytic acid chelates zinc effectively and it has a strong negative effect on zinc absorption in animal guts [155]. Improved irrigation can produce rice much healthier for farmers and their livestock. 

### 3.7. Effect of Animal Feed Management in Small Farms in Cambodia on Zinc Availability

Until very recently almost all small farmers have used untreated rice bran as a food supplement for their cows, pigs, ducks, chickens and fish. In visits to >100 farms in Kandal, Pursat, Kampong Cham, and Battambang provinces, Cambodia, commercial feeds were never observed to be used for livestock [96]. In Preak Russey, rice bran has 1.8 times and 2.1 times the concentration of zinc as brown rice and polished rice, respectively [96]. But rice bran has about 3.4 to 4.8 times the phytic acid content of brown rice or polished rice, respectively [156]. Rice bran contains about 86% of the phytic acid of the rice grain. Phytase is an enzyme produced by microbes, including those in some animal stomachs, but not much in humans, that can biodegrade phytic acid [157]. In many animals, the bacteria in the stomach do not produce optimal levels of phytase for zinc assimilation. In developed countries, phytase is purchased from agricultural vendors and included in animal feeds. Phytase is available from several vendors in SE Asia. In May 2021, one highly advanced agricultural feed supplier, began supplying animal feeds with phytase and other enzymes to improve the nutrition of livestock in Cambodia [158]. Using untreated rice bran especially from the arsenic zone is not a good farm management practice. Farmers need advice to manage phytic acid and zinc deficiency ideally mostly using their farm products. 

In Europe and North America one of the most common reasons for adding phytase to animal diets is to release phosphorus from phytic acid, and in turn decrease the amount of phosphorus that must be added in the animal diet [159]. This also reduces the amount of phosphorus in animal wastes which can reduce eutrophication of water bodies. Babatunde et al. [160] observed that phytase added to chicken diets improved the growth, nutrient and mineral digestibility, and bone mineralization of birds. Many of the papers on phytase in animal feeds were primarily concerned that the enhanced growth of the chicken was undertaken in a cost-effective manner. At least, in simple procedures, Sommerfeld et al. [161] reported that phytase addition to the diet did not completely degrade phytic acid. 

Chickens or ducks would be appropriate animals to evaluate the effect of improved zinc nutrition in an experimental fashion. Relative to cows, in particular, they are inexpensive. It is easy to monitor their growth, blood zinc levels, egg production and zinc content of the eggs. Reducing disease burden is an important objective but that is more complicated to assess. 

Management of other Cambodian livestock also needs improvement. Bhanderi et al. [162] stated that the diet of most cows in India was deficient in trace metals including zinc and proposed supplementation of their diet to improve resistance to disease. When zinc deficiency is recognized, it is common in more advanced countries for farmers to either add zinc to pastures or to provide salt licks that contain essential minerals and micronutrients for animals [163]. Cows in Preak Russey looked extremely gaunt (Figure 4). They might have suffered from arsenic toxicity or zinc deficiency but they might also suffer from various diseases or parasites, perhaps aggravated by arsenic toxicity or zinc deficiency. It would be useful to compare the levels of arsenic and zinc in blood from cows from this extreme site to cows in a control site such as Battambang in NW Cambodia which is outside the arsenic zone to confirm this condition. Although the main theme of this paper is viral pandemics, bacterial diseases should be considered with the same concern, including their potential to enhance viral disease, comorbidity.

Cows and people are susceptible to melioidosis which is caused by the bacterium *Burkholderia pseudomallei* that is commonly present in soils in SE Asia. The cows in Preak Russey (Figure 4) should be analyzed for melioidosis. Cows are less sensitive to melioidosis than people and cows are the vector spreading the disease between fields. Analysis of *Burkholderia pseudomallei* is possible with PCR [164] and other methods [165]. Melioidosis is often mistakenly identified as tuberculosis [166]. *Burkholderia pseudomallei* infects lungs when animals inhale or ingest dust. The cows in Preak Russey, especially in the dry season, graze on rice paddies irrigated with arsenic rich water. Mortality for people from melioidosis varies globally from 9% to 70% [165]. Data on melioidosis in Cambodia are becoming more available; some provinces, especially Takeo have much more melioidosis, but factors influencing its distribution are not well documented in Cambodia [167]. Most people are not aware of melioidosis and farmers take no precautions to avoid such infection [168]. Immunosuppression enhances melioidosis [165]. Arsenic toxicity and zinc nutrition could influence the distribution of melioidosis. 

### 3.8. Concerns with Arsenic in Aquaculture—An Important Source of Zinc

Aquaculture is an essential aspect of Cambodian agriculture. Fish are an important source of zinc [143] and affordable sources of zinc are required to prevent disease. Furthermore, fish are an important source of omega-3 fatty acids which are also important for immune systems [125]. There is nothing known in aquaculture equivalent to the Nipah virus or avian influenza but aquaculture is closely linked to terrestrial agriculture and human health and aquaculture should be part of an ecosystem analysis to improve management of pandemics. Aquaculture in Cambodia is a relatively new undertaking. The effects of arsenic in fish farms in Cambodia are undocumented. We expect that arsenic will enhance fish disease and exacerbate microcystins and geosmin, two important problems. Relative to livestock, aquaculture is much less likely to be a direct cause of a pandemic but the following aspects in the management of aquaculture are critical to human health and are linked closely to general agriculture: clean water availability, arsenic, rice bran, phytase, and intestinal microbiology. In Cambodia, in 2016, 80% of fish food was composed of rice bran [169]. Aquaculture is evolving quickly but the use of rice bran is still common [170]. In Preak Russey, rice bran had total arsenic levels (545 ± 134 µg/kg) that were 3.5 and 2.4-fold richer in inorganic arsenic than white or brown rice, respectively. The total arsenic in Preak Russey rice bran was 91% inorganic arsenic [96]. There will be arsenic residues in the fish flesh grown with rice bran, but it is likely that most of the arsenic will be organic which is much less toxic [171]. In Taiwan, to reduce adverse effects on human health, Jang [172] recommended that “groundwater utilization should be reduced substantially or even prohibited completely” in fish farms in areas with high arsenic concentrations in groundwater. Because of rice bran, especially in the arsenic zone, Cambodia might have a more serious situation than Taiwan. The effluent from some Cambodian fish farms will be enriched with arsenic. An analogous situation existed in the USA where chicken feed contained Roxarsone, an organic arsenic that suppressed parasites in chickens. Roxarsone breaks downs to inorganic arsenic. The downstream effect of inorganic arsenic from chicken farms was enough of a concern that USEPA banned the use of Roxarsone [173].

Arsenic is known to influence the microbial composition of soils [174]. The microbial composition of pond sediments and water will influence production of toxic algae and taste and odour compounds like geosmin. The role of micronutrients to control the composition of microbial communities has been well known for iron for almost five decades [175]. In humans during infections, the concentrations of iron and zinc in the blood plasma are reduced in part to control pathogens [96,176]. Geosmin and 2-methylisoborneol (MIB) are produced by microbes and result in nasty odour and taste of fish. The only effective method of reducing these taste and odour compounds has been to move the fish into clean water for two weeks prior to their sale [177]. Recently the production of geosmin and MIB by *Streptomyces* has been shown to be part of a mutualistic relationship with some invertebrates, springtails [178]. The springtails are attracted to the strong odour of geosmin and MIB and then disperse the spores of *Streptomyces*. *Streptomyces* also produces antibiotics to suppress other invertebrates; these *Streptomyces* antibiotics are regulated by concentrations of zinc. Moreover, zinc can block the production of geosmin by *Streptomyces* [179]. Recently a vendor is advertising that their fish-food supplement composed of a single-cell protein microbe with immunonutrients significantly reduces the concentration of geosmin and MIB [180]. The immunonutrient could be zinc and the single cell microbe could act like a probiotic and physically block *Streptomyces* or other producers of geosmin and MIB from being adsorbed into fish. Lukasse et al. [181] documented that geosmin mainly enters fish via their guts and proposed that probiotics in fish feeds should block geosmin bioaccumulation. Many probiotic treatments are used in aquaculture to change the microbial communities in animal guts to break down antimetabolites like phytase and to improve immunity [182]. 

Zinc deficiency has been shown to increase the sensitivity of Pangasius, an important fish in Cambodian aquaculture, to lead toxicity, heat and fish disease [183]. Climate change will enhance heat stress. Lead and arsenic both stress fish via oxidative stress [183,184]. Similarly, zinc deficiency had been shown to enhance arsenic toxicity in zebrafish [185]. Lin et al. [186] found that zinc supplementation to shrimp ponds increased the growth and immune systems of shrimp. Currently fish farmers use various pharmaceuticals to control fish disease but there are guidelines and concerns about pharmaceutical residues left in fish [169]. The use of rice bran without zinc supplementation for fish food is likely to result in zinc deficiency and perhaps in turn, weakened immune systems, enhanced sensitivity to heat and fish disease. In Thailand, corn is the main ingredient in fish food [187]. In the USA, when corn is deficient in zinc, the corn is enriched with zinc prior to being fed to the fish [188]. Recently soybeans have been imported from the USA to produce fish food in Cambodia [189]. Because rice is the only major crop grown under sustained flooding, rice can be ten times as contaminated with arsenic as corn or other grains [190,191]. Soybeans and corn are much better selections than unprocessed rice bran from the arsenic zone of Cambodia or Vietnam for either fish or livestock food. 

## 4. Conclusions

In order to increase the resilience of the Cambodian population, and the world from pandemics, it would be prudent to conform with guidelines recommended by the WHO, USA-EPA, EU, ASEAN or similar agencies for arsenic, mercury, and zinc. In Cambodia, guidelines are not always consistent with international agencies or as with irrigation water do not exist at all (Table 2). Based on our more detailed discussions above, we also summarize possible remedial actions for Cambodia to improve human health and enhance resistance to new pandemics (Table 3).

Pandemics will arise again and potentially be worse than the COVID-19 pandemic. There have been human mortalities of 40% with the Nipah virus [63] and human mortalities of 39% and 53% with two separate outbreaks of avian influenza [69]; these three virus outbreaks had mortality rates >780 times that of the COVID-19 outbreak (24 June 2021) [13]. We must prepare. Control of trapping, shipping and eating of wild game should be enhanced. Improving human health with better nutrition and avoidance of toxins should have high priorities. Adapting lifestyles with effective policy and strong education would augment ongoing nutrient supplementation plans [198]. Innovative solutions are required to improve micronutrient nutrition, i.e., school feeding programs [7]. Agricultural methods should improve to produce food with more bioavailable zinc. As with many other countries, toxins like arsenic and mercury are well known to impair human health but yet they remain problems in Cambodia. It is possible that with appropriate education, the ongoing anxiety with the COVID-19 pandemic could motivate improved toxin management. Arsenic and mercury suppress the immune system and excessive exposure must be avoided to reduce the virulence of pathogens. 

## Figures and Tables

**Table 1:** The guidelines for arsenic in irrigation water (µg/L).

Country	EU [117]	Canada [116]	Australia [115]	Taiwan [121]	S. Korea [120]	Italy [119]	Japan [118]
Arsenic	100	100	100	50	50	20	10

**Table 2:** Suggested guidelines for better environmental health.

Parameter	Suggested Guideline Reference			Current Khmer
	Authority	Guideline	Reference	
As drinking water	WHO	10 µg/L	[99]	50 µg/L
As irrigation water	South Korea	50 µg/L	[120]	none
Inorgan As-general	Codex	200 µg/kg	[192]	200 µg/kg
Inorgan As-children	EU	100 µg/kg	[193]	none
Zn rice children	Codex	14.7 µg/g	[194]	none
Ing-Hg diet	USEPA	0.3 µg/kg-d	[195]	none
Me-Hg-diet	USEPA	0.1 µg/kg-d	[196]	none
Hg Cosmetics	ASEAN	1 µg/g	[197]	1 µg/g

WHO World Health Organization, Codex regulations with The World Trade Organization; EU is European Union, USEPA is United States Environmental Protection Agency, ASEAN is the Association of Southeast Asian Nations. As is arsenic. Inorgan As is inorganic arsenic. Zn is zinc. Ing-Hg is inorganic mercury. Me-Hg is methyl mercury.

**Table 3:** Suggested actions for better disease management.

Block sales of wildlife to reduce export of animal diseases
Develop bioassays for potential new pandemics like Nipah virus, avian flu to select therapeutics
Treat groundwater to remove arsenic to less than 50 µg/L
Improve irrigation supplies to allow AWD # irrigation, produce better quality rice
Encourage new crops for dry season using less water and be more resilient to climate change
Add phytase to animal feeds to make zinc more bioavailable
Add zinc and other needed micronutrients to animal feeds
Continue developing post-harvest nutrient supplementation including in schools
Resolve if inorganic arsenic bioaccumulates in aquaculture in the arsenic zone
Develop fish consumption guidelines for mercury
Improve artisanal gold mining to avoid use of mercury

# AWD alternative wet dry.

## Data Availability

All publications cited and published by this team are availability in Researchgate.
